# Protocol: An absolute egg-to-adult viability assay in *Drosophila melanogaster*

**DOI:** 10.17912/micropub.biology.001656

**Published:** 2025-08-21

**Authors:** Cassidy Schneider, Spencer Koury, Laurie S. Stevison

**Affiliations:** 1 Biological Sciences, Auburn University, Auburn, Alabama, United States

## Abstract

Measures of organismal fitness must take into account reproductive output and survivorship across life-history stages. In *Drosophila melanogaster* , a laboratory model system, these traits are often quantified with egg-laying assays and egg-to-adult viability. While several protocols for automated egg counting exist, these methods typically preclude directly analyzing phenotypic distributions in resulting adults. Here, we digitally score eggs from photographs taken under standard laboratory culturing conditions (cornmeal media in polystyrene vials) using a method compatible with scoring surviving adults. This absolute measurement of egg-to-adult viability, that also allows investigation of survivorship biases, was applied to three commonly used laboratory strains.

**Figure 1. Methodology and Validation.**
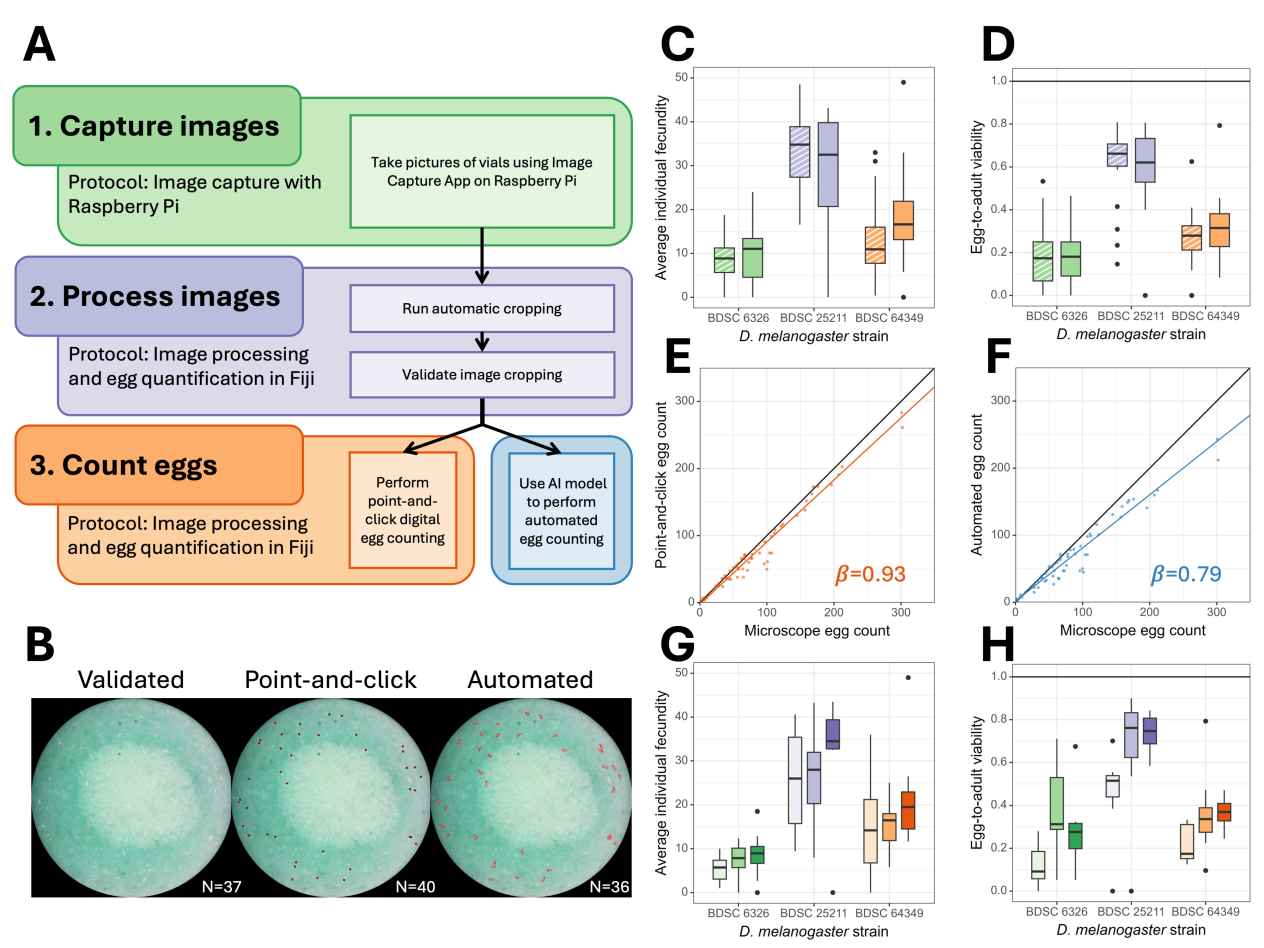
(A) Egg quantification workflow where subtitles correspond to protocol associated with that step. (B) A representative image of blue food egglaying surface that has been processed to prepare for counting (left), manually annotated using a point-and-click approach (middle), and as predicted by an AI image segmentation model on arivis Cloud (right). The N denotes the number of eggs detected either by microscope manual count (left), point-and-click (middle), or automated (right). (C) Average daily individual fecundity for each of three strains: *BDSC 6326* (green), *Canton-S* (purple), *Oregon-R* (orange) do not differ between vials prepared with regular food (striped) and those with blue food dye added (solid) **( *F _1,132_ = 0.063, p = 0.80* ),** where average individual fecundity is the number of eggs laid divided by the parental female density reported on a per vial basis. (D) Egg-to-adult viability for each of the same three strains similarly color coded, measured for each vial by dividing the number of surviving adults by the number of eggs **( *F _1,125_ = 0.057, p = 0.81* )** . (E) Comparison of manual egg counts using a dissecting microscope to counts obtained using the point-and-click protocol on digital images with model II regression coefficient reported ( *95% CI: 0.89 - 0.97* ). (F) Comparison of manual egg counts to automated counts obtained using an image segmentation model with model II regression coefficient reported ( *95% CI: 0.74 - 0.84* ). (G) Comparisons of individual fecundity between the same three strains, across three successive 24-hour broods (A, B, C) indicated by increasing color intensity. (H) Comparisons of egg-to-adult viability across the same three strains and same three brooding periods.

## Description

The logistical difficulty in measuring Darwinian fitness, even in a laboratory setting, means that fitness components such as survivorship, mating success, or fecundity are commonly used as surrogate measures (Prout 1971; Mukai et al. 1974; Fowler et al. 1997; Crone 2001; Flatt 2020; Wadgymar et al. 2024). Here, we focus on egg-to-adult viability (defined as the proportion of zygotes that survive to adulthood) as a measure of survival in the genetic model system *Drosophila melanogaster.* Genetic inference using experimental crosses of *D. melanogaster* benefits from ~80,000 strains that include balancer chromosomes and extensive, publicly available mutant stocks (Ashburner et al. 2011; Hales et al. 2015; Bilder and Irvine 2017). However, many of the available mutant phenotypes are restricted to the adult life-history stage and have known viability defects (Hurst 2019; Öztürk-Çolak et al. 2024). Not accounting for experimental death prior to the adult stage can have major implications on inferences that rely heavily on distribution of adult phenotypes or mutant markers (Gilchrist and Partridge 1997). This is a long-standing and well-known problem in recombination studies (Bailey 1962), but also extends more broadly to regression-based heritability estimates, developmental perturbations, and any study where biases in survivorship across generations or life-history stages will confound analyses.

Absolute egg-to-adult viability requires quantification of both eggs in a vial and adults surviving to eclosion. Scoring adult flies under laboratory culturing conditions is highly standardized in the over 3000 Drosophila labs worldwide (Bilder and Irvine 2017), so our efforts focus on egg quantification in a method that does not alter the measurement of adults or survival probabilities. While there are several existing protocols for egg quantification that are primarily aimed at quantifying fecundity (Waithe et al. 2015; Ng’oma et al. 2018; Nouhaud et al. 2018; Gomez et al. 2024), methodological constraints sometimes do not allow for completion of development or the full life cycle. Specifically, assays using grape juice media in petri dishes, 96-well plates, or that freeze eggs for quantification severely limit the potential to measure egg-to-adult viability (Elgin and Miller 1980; Ashburner 1989; Gomez et al. 2024).

Here, we developed an approach to measure absolute egg-to-adult viability that could be performed with common Drosophila culturing conditions such that the experimental organisms could complete normal egg-to-adult development and those adults could be phenotypically scored (see Methods). Briefly, we provide an Image Capture App for Raspberry Pi to take digital pictures of eggs on the surface of high contrast food (created by 0.1% v/v blue food coloring) following a 24-hour egg-laying period. The images are then processed using Fiji, preparing them for egg quantification by the methods described here. The egg quantification workflow is illustrated in Figure 1A with example pictures showing scored eggs in Figure 1B . Finally, the eclosing adults are scored to calculate the proportion of zygotes that complete development which allows assessment of any survivorship bias in that observable fraction of offspring. To evaluate this egg-to-adult viability assay, we conducted an experiment to measure fecundity and egg-to-adult viability from three commonly used, highly inbred, laboratory stocks of *D. melanogaster* (RRID:BDSC_6326 “ *w ^1118^* ”, RRID:BDSC_25211 “ *Oregon-R* ”, and RRID:BDSC_64349 “ *Canton-S* ”).

First, we examined the effects of the modified Drosophila culturing conditions ( *i.e.* addition of blue food coloring). Using manual egg counts (performed by SK under 10-20x magnification on a dissecting microscope), daily individual fecundity ( *DIF _ijkl_* ) was measured as number of eggs laid per female per 24 hours and egg-to-adult viability ( *EAV _ijkl_* ) measured as number of eclosing adults divided by the number of eggs laid in that vial. These response variables were analyzed with an ANOVA based on the following linear models:

*DIF _ijkl_ = B _i_ + S _j_ + M _k_ + e _ijkl_* , equation 1

*EAV _ijkl_ = B _i_ + S _j_ + M _k_ + e _ijkl_* . equation 2

Where both response variables are a linear function of the *i ^th^* brood effect ( *B _i_* ), the *j ^th^* strain effect ( *S _j_* ), the *k ^th^* fixed media effect ( *M _k_* ), and a random error term ( *e _ijkl_* ). ANOVA tables for these linear models treat both brood and strain as blocking effects, reported in tables 1 and 2 of the extended data, respectively. This analysis reveals that the addition of 0.1% v/v blue food coloring to the standard cornmeal Drosophila media has no effect on daily individual fecundity ( *F _1,132_ = 0.063, p = 0.80* ) ( Figure 1C ) and no effect on egg-to-adult viability ( *F _1,125_ = 0.057, p = 0.81* ) ( Figure 1D ). Therefore, all subsequent analyses only consider data collected from vials with 0.1% v/v blue food coloring that provides high contrast for translucent eggs on the blue food background.

Second, we investigated the correlation among the different methods of egg quantification; namely, microscope egg counts, point-and-click egg counts, and automated egg counts by AI. These measures were made by two different authors (SK and CS) The overall correlation between methods on blue colored media was very strong (Pearson correlation coefficients *r = 0.97* for automated versus microscope counts, *r = 0.98* for point-and-click versus microscope counts, and *r = 0.99* for automated versus point-and-click counts). However, regressing the two different types of digital egg counts on manual microscope egg counts with a model II major axis regression yields regression coefficients that are significantly below the 1:1 line; *b = 0.93, 95% CI: 0.89 - 0.97* for point-and-click versus microscope counts ( Figure 1E ) and *b = 0.79, 95% CI: 0.74 - 0.84* for automated versus microscope counts ( Figure 1F ). This suggests that the digital images contain information that the AI model is not yet sufficiently able to detect, and that although the different methods of egg counting produce highly correlated results, the automated egg count is not accurate in an absolute sense and is consistently undercounting the total number of eggs in a vial by about 20%. Two avenues to address this problem would be to either use a statistical model to apply a bias correction to automated egg counts (Waithe et al. 2015) or alternatively to revisit the automated egg counting procedure as AI models improve. Here, all subsequent analyses are based only on point-and-click counts that represents a balance of scalable digital methods and high-fidelity accuracy for egg quantification under standard Drosophila culturing conditions.

Third, we demonstrate the intended use of the protocol by using the ANOVA to partition variation between genotypes and broods of daily individual fecundity and egg-to-adult viability. This procedure has the added benefit of quantifying the repeatability of these measures and estimating the respective mean squared errors which the reader can use in their own experimental design and power analysis (see extended data). The linear models for daily individual fecundity ( *DIF _ijk_* ) and egg-to-adult viability ( *EAV _ijk_* ) using high contrast blue colored food are:

*DIF _ijk_ = B _i_ + S _j_ + e _ijkl_* , equation 3

*EAV _ijk_ = B _i_ + S _j_ + e _ijkl_* . equation 4

Where the right-hand side of both equations are the *i ^th^* brood effect ( *B _i_* ), the *j ^th^* strain effect ( *S _j_* ), and a random error term ( *e _ijk_* ). Inspection of the sum of squares in the ANOVA tables 3 and 4 (see extended data) shows that in both traits, irrespective of statistical significance, the brood effect accounts for less than 10% of total phenotypic variation and greater than 50% of phenotypic variation is unexplained. There are clear statistically significant effects of strain on both daily individual fecundity ( *F _2,64_ = 26.43, p = 4.28x10 ^-9^* , Figure 1G ) and egg-to-adult viability ( *F _2,63_ = 25.70, p = 6.90x10 ^-9^* , Figure 1H ) which account for 43% and 41%, respectively, of the total phenotypic variation in our experiments.

The overall egg-to-adult viability was 35%, a surprisingly low number for the relatively benign environment of standard Drosophila culturing conditions. Some fraction of eggs in our assay may be unfertilized. Indeed, unmated females from some strains can lay prodigious numbers of eggs that will produce no zygotes (Boulétreau-Merle et al. 1989) and any unfertilized eggs would lower our viability estimate. Although we did not explicitly investigate this, some experiments may be interested in this fraction for biological reasons, which can be directly estimated from standard petri dish egg-laying assays run in tandem ( *e.g.* , Sheahan et al. 2023). Despite the variability in our measures (>50% of phenotypic variation is unexplained in both), it is still possible to detect (with statistical significance) differences among crosses, genotypes, or even environmental perturbations. Implementation of this assay demonstrates that absolute viability is a critical measure when conducting genetic experiments where inferences are potentially confounded by survivorship bias across generations or life-history stages. Furthermore, the magnitude and variation in experimental mortality across strains means that the choice and control of genetic background ( *i.e.* strain) is a primary concern on par with sample size and statistical power considerations.

## Methods

Our protocol has been modularized for ease and clarity. Specifically, the protocol is composed of three separate protocols that cover (1) food preparation (2) image capture using a Raspberry Pi (associated GitHub repository: https://github.com/StevisonLab/ImageCaptureApp ), and (3) image processing using Fiji (associated GitHub repository with raw data: https://github.com/StevisonLab/Egg-to-adult-viability ), which includes egg counting using a point-and-click approach. A workflow implementing the latter two protocols is depicted in Figure 1A . A static release of each repository is included as Extended Data.

The collection of protocols described in this peer-reviewed article is published on protocols.io, dx.doi.org/10.17504/protocols.io.4r3l264qqv1y/v1. The individual protocols are published on protcols.io (Food preparation: dx.doi.org/10.17504/protocols.io.4r3l2pjnxg1y/v1, Image capture with Raspberry Pi: dx.doi.org/10.17504/protocols.io.261geeoywg47/v1, Image processing and egg quantification using Fiji: dx.doi.org/10.17504/protocols.io.q26g75bo3lwz/v1). A static release of this collection is included as Extended Data.

To demonstrate the utility of our protocol, we conducted an experiment to measure fecundity ( Figure 1G ) and egg-to-adult viability ( Figure 1H ) from three commonly used highly inbred, laboratory stocks of *Drosophila melanogaster* obtained the from the Bloomington Drosophila Stock Center (RRID:BDSC_6326 “ *w ^1118^* ”, RRID:BDSC_25211 “ *Oregon-R* ”, and RRID:BDSC_64349 “ *Canton-S* ”). For each of the three stocks, two replicated crosses were conducted at female by male densities of 1x1, 2x2, 3x3, 4x4, 5x5, 6x6, 7x7, and 8x8 for a total of 46 crosses (insufficient virgins were present to conduct the two 8x8 *Oregon-R* crosses). All flies were reared in typical laboratory conditions (21°C, 70% relative humidity with a 12-hour light-dark cycle) and cultured in narrow polystyrene vials (Cat #: 32-109, Genessee Scientific) on standard cornmeal Drosophila media (Bloomington Drosophila Stock Center Cornmeal Food Recipe). Adult females were collected as virgins over a 72-hour period, aged for an additional 72-hour period, and then crossed with similarly aged males from the same strain under light CO _2_ anesthesia. After a 24-hour CO _2_ recovery period, each set of mated pairs were tap transferred onto fresh food for three successive 24-hour egg laying periods, hereafter referred to as broods.

To heighten contrast of eggs against the standard cornmeal media, McCormick blue food dye was added to the fly food recipe in a 0.1% volume by volume concentration (Protocol #1). To induce maximal egg-laying behavior (Becher et al. 2012), without compromising ability to digitally image eggs, 50 microliters of a 0.2 g/mL yeast paste with 0.2% volume by volume blue food dye was directly aliquoted into the center of each vial. Optimal concentrations, volumes, and timings were determined by a series of pilot studies where we experimented with various parameter combinations, while avoiding the published density dependent relationship between number of eggs and egg-to-adult survival (Gardner et al. 2001; Sharp and Agrawal 2008; Kristensen et al. 2015). We used a Nikon SMZ645 Stereo Microscope to manually count eggs at 10x total magnification for validation of the egg counts via images.

We took pictures of eggs in vials using a 5mp camera with an M12 mount (SKU B0031, Arducam) with a lens with a 10° HFOV (SKU LN001, Arducam). Similar to published image capture setups, images were captured in the dark, using lamps to light the vial for imaging. We used 2 lamps with bright white bulbs at 12.2W, 1100 lumens, daylight (5000K) bulbs (Model # BR30DMHO/950CA/2, Feit Electric) pointed directly up to provide the best illumination and minimize glare. We used a 3D printed vial holder (available as “VialHolder.stl” at https://github.com/StevisonLab/Egg-to-adult-viability ) to block light entering vial from the side below the egg-laying surface.

We controlled the camera and took the pictures with a custom desktop application for Raspberry Pi (available at https://github.com/StevisonLab/ImageCaptureApp). This application allows the user to input a list of vials and then click a button to take a picture for each one (Protocol #2). The raw images were 2592px by 1944px and were saved as PNGs to avoid compression artifacts.

The images were processed and analyzed using the Fiji platform (Schindelin et al. 2012) as outlined in protocol 3 . The scripts used to perform these steps are available at https://github.com/StevisonLab/Egg-to-adult-viability ). To prepare the images for downstream quantification, a Hough circle transform was performed using the IJ-OpenCV library (Domínguez et al. 2017), which connects Fiji to the computer vision library OpenCV (Bradski 2000). The vial image was cropped to the square that bounds the circle, and the area of the image not contained in the circle was deleted (pixels set to black; Figure 1B ). This cropping was validated and adjusted as needed. Once the image processing was complete, the provided script was used to count the eggs by clicking to mark them, referred to as “point-and-click” (middle of Figure 1B ).

We also used the arivis Cloud platform (Carl Zeiss Microscopy, 2023) to train an AI model to detect eggs by outlining eggs on pictures of vials. The model was trained on images taken of vials of various genotypes and various treatments at varying densities during the development of the assay. After training, the model was used to detect eggs in new images. The total number of predicted egg objects was extracted from the output using a custom script, an illustration of which is presented in the right panel of Figure 1B .

Finally, we kept the vials where eggs were laid until counting all the adult flies that emerged from pupae. We compared the emerging adult counts to the egg counts for each vial to obtain a measure of egg-to-adult viability. Total egg-to-adult viability was calculated by dividing the number of surviving adults by the number of eggs. All data was analyzed using R (R Core Team 2025). Pearson correlation coefficients were calculated using base R function ‘cor’, linear models were fit using ordinary least squares with function ‘lm’, and model II major axis regression coefficients were calculated using R package ‘lmodel2’ (Legendre and Oksanen 2024). For visualization, we used the ‘ggplot2’ package (Wickham 2011). Full ANOVA tables can be found in extended data.

## Reagents

**Fly strains used:**

**Table:** 

**Strain**	**Species**	**Resource Reference ID (RRID)**	**Available From**
**w1118**	*Drosophila melanogaster*	BDSC_6326	Bloomington Drosophila Stock Center
**Oregon-R**	*Drosophila melanogaster*	BDSC_25211	
**Canton-S**	*Drosophila melanogaster*	BDSC_64349	

**Protocol #1: Fly Food with Blue Food Dye**

· Fly vials: Polystyrene narrow vials for Drosophila from VWR (75813-162)

· Vial Plugs: Drosoplugs for narrow vials from Genesee Scientific (59-200)

· Soy flour: FlyStuff NutriSoy Soy Flour from Genesee Scientific (62-115)

· Agar: Drosophila Agar Type II 80/100 Mesh from Genesee Scientific (66-103)

· Corn meal: Yellow Corn Meal from Genesee Scientific (62-100)

· Corn syrup solids: Tate & Lyle Corn Syrup Solids from Genesee Scientific (62-109)

· McCormick Blue Food Color: 43217-41014

· Tegosept: p-hydroxy benzoic acid methyl ester from Genesee Scientific (20-258)

· Propionic acid: Propionic Acid Sodium Salt from Genesee Scientific (20-271)

**Protocol #2: Image Capture with Raspberry Pi:**

· Raspberry Pi (Model 4B) set up for taking pictures ( https://github.com/StevisonLab/ImageCaptureApp )

· 5mp camera (SKU: B0031, Arducam) 1/4” (OV5647)

· 1/2.5″ M12 Mount 16mm (SKU: , Arducam)

· M12 lens with HFOV 10° on 1/4” RPi Camera (SKU: LN001, Arducam)

· Vial holder ( https://github.com/StevisonLab/Egg-to-adult-viability/blob/main/VialHolder.stl )

· 2 lamps with 12.2W, 1100 lumens, daylight (5000K) bulbs (e.g., Model # BR30DMHO/950CA/2, Feit Electric)

· Tray of vials to be photographed

· *Optional* . Empty tray (for completed vials)

**Protocol #3: Image processing for egg quantification**

· Software setup: https://github.com/StevisonLab/Egg-to-adult-viability

## Data Availability

Description: Statistical model tables for all analyses described.. Resource Type: Text. DOI: https://doi.org/10.22002/p87d1-zm421 Description: Image Capture App GitHub Repository. Resource Type: Software. DOI: https://doi.org/10.22002/ph5wb-6rd20 Description: Image Processing GitHub Repository. Resource Type: Software. DOI: https://doi.org/10.22002/1yz9y-12x13 Description: Associated Protocols published on Protocols.io. Resource Type: Workflow. DOI: https://doi.org/10.22002/fgaes-75d66
